# Reporting on the establishment of a Privacy Preserving Record Linkage to Facilitate an Ongoing Crosswalk Between Research and Health Administrative Databases

**DOI:** 10.23889/ijpds.v9i5.2761

**Published:** 2024-09-18

**Authors:** Mike Jarrett

**Affiliations:** 1 Population Data BC

## Introduction

Data linkage centres receive, hold and provision data in a wide variety of formats. Using a consistent, flexible and open metadata standard for data holdings can enable a center to clearly and unambiguously describe their data holdings and facilitate processing pipelines. One such framework is the Frictionless data standard. We present our experience at our data centre implementing this standard for both internal data management and for communication with external data providers and third parties.

## Results

Our team has replaced tool-specific metadata files with standard-compliant files to make our pipelines more modular and less fragile. Since the standard is based on JSON, most programming languages will support reading and writing metadata files and we are not restricted to any software. This work has also made our internal tooling interoperable with that of external partners. We find that the standard is well equipped to handle CSV and other delimited files. Our center uses a mix of file types including fixed-width text files which do not have an explicit specification within the standard, but its extensibility has allowed us to define our own file type while staying within the ecosystem. We have also developed tools to convert JSON metadata files to excel workbooks for a more human-friendly format.

## Results

The Frictionless data standard can be a powerful tool for data centers in organizing data and building processing pipelines. 

## Implications

Adopting the Frictionless data standard for metadata files has streamlined our internal processes and improved internal and external communication.

